# The spatio-temporal dynamics of infant mortality in Ecuador from 2010 to 2019

**DOI:** 10.1186/s12889-022-14242-1

**Published:** 2022-10-01

**Authors:** Karina Lalangui, Karina Rivadeneira Maya, Christian Sánchez-Carrillo, Gersain Sosa Cortéz, Emmanuelle Quentin

**Affiliations:** 1 Centro de Investigación EpiSIG, Instituto Nacional de Investigación en Salud Pública, Quito, Ecuador; 2 Ministerio de Salud Pública, Quito, Ecuador; 3 Facultad de Geografía, Universidad Autónoma del Estado de México, Toluca, México; 4 Centro de Investigación en Salud Pública Y Epidemiología Clínica (CISPEC), Universidad UTE, Quito, Ecuador

**Keywords:** Infant mortality rate, Spatio-temporal analysis, Spatial clusters, Time trends, Ecuador

## Abstract

The infant mortality rate (IMR) is still a key indicator in a middle-income country such as Ecuador where a slightly increase up to 11.75 deaths per thousand life births has been observed in 2019. The purpose of this study is to propose and apply a prioritization method that combines clusters detection (Local Indicators of Spatial Association, LISA) and a monotonic statistic depicting time trend over 10 years (Mann–Kendall) at municipal level. Annual national databases (2010 to 2019) of live births and general deaths are downloaded from National Institute of Statistics and Censuses (INEC). The results allow identifying a slight increase in the IMR at the national level from 9.85‰ in 2014 to 11.75‰ in 2019, neonatal mortality accounted for 60% of the IMR in the last year. The LISA analysis allowed observing that the high-high clusters are mainly concentrated in the central highlands. At the local level, Piñas, Cuenca, Ibarra and Babahoyo registered the highest growth trends (0.7,1). The combination of techniques made it possible to identify eight priority counties, half of them pertaining to the highlands region, two to the coastal region and two to the Amazon region. To keep infant mortality at a low level is necessary to prioritize critical areas where public allocation of funds should be concentrated and formulation of policies.

**Supplementary Information:**

The online version contains supplementary material available at 10.1186/s12889-022-14242-1.

## Background

Infant mortality (IM) remains a serious global public health problem [1, 2], not all infants under one year of age have the same opportunities to enjoy survival [3]. Biological, socioeconomic, environmental and care determinants are among the main risk factors for IM [4–6]. However, most deaths are preventable and treatable. Globally, approximately 70% of infant deaths are due to preventable causes [7], especially inadequate health care for pregnant women and newborn care [8].

One of the most widely used indicators to measure health status and human development is the IMR [9, 10], defined as the number of deaths of children under 1 year of age per 1,000 live births in the same year [11]. The global IMR has declined markedly, it decreased from 65‰ in 1990 to 29‰ in 2019 [12]. In the Americas, the countries that make up the Andean region have also reduced the IMR, Ecuador recorded 42.2‰ in 1990 and 11.6‰ in 2019 [13, 14], while neighboring countries, specifically Colombia went from 29.2‰ to 11.7‰ and Peru from 56.7‰ to 10.3‰ in the same years [13, 14]. However, the pace has been slow compared to other regions such as North America and the Southern Cone [13], another concern is that it is uneven across regions and socioeconomic groups [15].

In public health, Geographic Information Systems (GIS) and spatial analysis have been used for epidemiological and health research [16]. MI has been approached from the spatial and temporal view in the United States, Mexico and Brazil [17–19], spatial thinking allows understanding the relative locations of complex social, environmental and demographic interactions that produce patterns of disease and death [19], also mapping the spatial distribution of IM can bring improvements to programs in terms of allocation of limited resources to those regions with high unmet health care needs [15].

In Ecuador, no studies have been found that use a spatial approach to understand the spatio-temporal dynamics of IM at the local level (municipality) and not only present national statistics. Therefore, this study proposes a method that combines techniques in spatial analysis to prioritize the critical areas where action should be taken to reduce IM. However other researches in Ecuador on suicide, cancer, and neglected tropical diseases have used significant spatial clustering to determine critical areas [20–22]. The methods used in this analysis have also been applied in other countries to locate spatial clusters, identify risk factors, and compare spatial variation in IM [15, 17, 23].

This study proposes a spatial analysis of IM in Ecuador at the level of municipalities and looks for areas where there are significant clusters below or above the national average. This could help to prioritize the sectors where greater accessibility and availability of child health services is needed. To prioritize areas for action, it is interesting to identify the municipalities where the highest rates are found and where the trend is strongly increasing. The main idea is to propose an innovative combination of available spatiotemporal techniques to support the required vigilance regarding IM.

## Methods

### Study area

Ecuador is located in South America, bordering Colombia (north), Peru (south-east) and the Pacific Ocean (west). Politically, it is divided into 24 provinces and 221 counties that correspond to municipalities or communes (second political-administrative level after provinces). It has four natural regions: coast, highlands, Amazon and Galapagos Islands. For this study only continental Ecuador was considered.

### Data source

The secondary databases of live births and general deaths are downloaded from the INEC website [24, 25]. The period covered is ten years from 2010 to 2019. The birth database for the study period includes all live births reported on birth certificates [24] and the death dataset includes all deaths of children under 1 year of age reported on death certificates [25] collected by each municipal civil registry from physical and digital forms of the National Vital Data Registry System.

### Data extraction

To apply a spatial study, the level of municipality (canton) is selected, for which the registrered record are counted in order to obtain the count of live births by canton of residence of the mother and the count of deaths of children under 1 year of age by canton of death (to preserve confidentiality, the residence does not appear in these databases). The records of non-residents in Ecuador are discarded since they won’t be mapped.

### Infant mortality rate

The formula applied is the following:

$$IMR=1000\times \frac{{deaths}_{<1 year}}{live\ births}$$

The yearly tables of IMR per 1000 live births by municipality allows to construct thematic maps.

### Time trend

The Mann–Kendall non-parametric statistical test is used to determine the time trend over a period of the annualized IMR. To apply this test, the data do not need to fit any particular distribution [26]. The statistic makes combinations of each pair of observed values, over time, that is, it checks whether *IMR*_*j*_ > *IMR*_*i*_ or *IMR*_*j*_ < *IMR*_*i*_ and counts the number of pairs that increase or decrease over time. It express the relative frequency of increases minus the relative frequency of decreases and it is calculated for each spatial unit as [27]:

$$S=\frac{2\left(t-2\right)!}{t!}\sum_{i=1}^{t-1}\;\sum_{j=i+1}^tsign\left({IMR}_j-{IMR}_i\right)$$

where the sign function is given by

$$sign\left({IMR}_{j}-{IMR}_{i}\right)=\left\{\begin{array}{c}1\ if \left({IMR}_{j}-{IMR}_{i}\right)>0\\ 0\ if\left({IMR}_{j}-{IMR}_{i}\right)=0\\ -1\ if \left({IMR}_{j}-{IMR}_{i}\right)<0\end{array}\right\}$$

IMR_i_ is the IMR in year $i\in \left\{\mathrm{1,2},\dots ,t-1\right\}$ with $t$ as the number of available years and IMR_j_ is the IMR in year $j=(i+1) \in \left\{\mathrm{1,2},\dots ,t\right\}$.

Mann–Kendall values range from -1 to + 1. When a value approaches + 1 it means there is a monotonic upward trend, when it approaches -1, the trend is downward and a value of 0 indicates no trend [28].

The Terrset software [28] has been used in order to apply this calculus.

### Spatial trend

The observed variable, in this case the IMR in the study area is represented with maps and using the spatial statistics technique for cluster detection using the Moran Indicator both globally and locally. The aim is to observe the spatial dependence that may or may not exist between nearby locations.

Considering a set of *N* spatial units in a region, the spatial autocorrelation represents the relationship between the IMR, in one spatial unit, and the IMR values of its n neighbors, which can be visualized through a connectivity map. To quantify the closeness between two spatial units, a positive *n x n* matrix *W* is used, made up of *n(n-1)* spatial weights called *wi,j* which are defined based on binary contiguity, like this [29]:

$${w}_{i,j}=\left\{\begin{array}{c}{w}_{i,j}=1\ if\ j\ne i,neighbouring\ space\ units\\ {w}_{i,j}=0\ opposite\ case\end{array}\right\}$$

The Moran Index (I) is the test considered to be the most applied and statistically strongest to detect spatial independence from debris, this being a summary measure of the intensity of the spatial association between units [29, 30]. Its range of values is based on the weight matrix, usually varying between -1 and + 1 but not necessarily restricted by this, unlike a correlation coefficient [31]. If its neighboring municipalities tend to have similar values in their IMR, *I* will be positive indicating that the pattern is clustered, if they are different, *I* will be negative, that is, the pattern is regular and when spatial randomness is present the expected value of *I* is given by *E[I]* = *(-1)/(n-1),* as *n* increases, *E[I]* approaches *0* [31].

Given *i* and *j* in {1,2,…,n}, the index is defined by:

$$I=\frac{n}{{\sum }_{i=1}^{n}\sum_{j=1}^{n}{w}_{i,j}}\frac{{\sum }_{i=1}^{n}\sum_{j=1}^{n}{w}_{i,j}\left({x}_{i}-\overline{X }\right)\left({x}_{j}-\overline{X }\right)}{{\sum }_{i=1}^{n}{\left({x}_{i}-\overline{X }\right)}^{2}}$$ for $$j\ne i$$,

where *n* is the total of municipalities, *x*_*i*_ the IMR in municipality *i, x*_*j*_ the IMR in another municipality *j,* $\overline{X }$ the average of the IMR and *w*_*i,j*_ the elements of the contiguity matrix *W* that links municipality *i* to *j*.

As there are spatial effects such as heterogeneity that refer to the indistinct behavior of the variable observed in each of the units, local patterns can be detected that with the global measure were ignored, so local measures are introduced as Local Spatial Association Indicators (LISA) is calculated as [32]:

$$I_i=\left(x_i-\overline X\right)\overset n{\underset{j=1}{\sum\;}}w_{i,j}\left(x_j-\overline X\right)forj\neq i$$

With this analysis, using the calculation of Moran's *I*_*i*_ and the scatter plot, four categories of groupings can be identified by the type of spatial association: the hotspots, which are municipalities with an above-average rate and the rate of their neighbors as well, the high-high category, or otherwise the below-average rate, the low-low category, and the outliers or atypical values, which are municipalities with an above-average rate but the rates of their neighbors are below the average, the high-low category, or otherwise the low–high category [33]. To see if these groupings were not created randomly, a statistic test of Moran is applied where the null hypothesis of randomness is opposed to the alternative of clustering, and the significance is obtained with a permutation approach. These techniques are available in the GeoDa software [33].

### Prioritization criteria for identification of spatial–temporal critical areas

Different types of criteria can be developed and implemented according to the prioritization needs of the study.

In this case, the methodology was designed according to logical criteria. First, in order to eliminate inconsistent rates, municipalities with less than 2 deaths were excluded. The counties with higher IMR during the most recent year were selected, using the 90% percentile threshold. The frequency, in number of year, of pertaining to a high-high or hotspot cluster is used to give priority. The third factor considered is the higher positive trend over all the period studied.

**Fig. 1:**
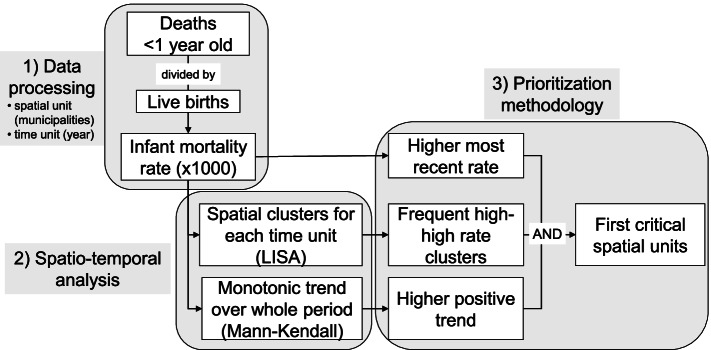
Methodology for data processing

Eventually the hotspot repetition over years can be more strictly evaluated using the logical AND operator instead of the OR operator (Fig. 1).

## Results

**Fig. 2:**
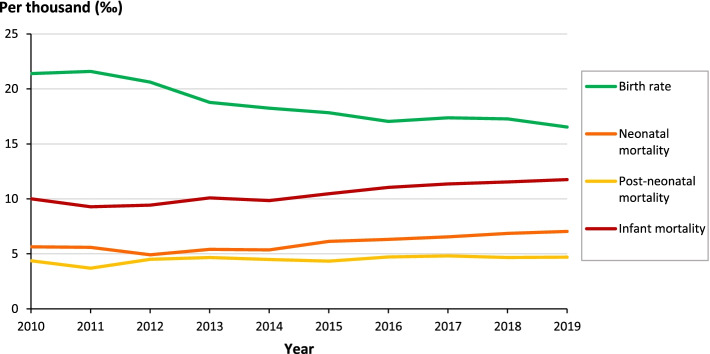
Yearly evolution of the national Infant Mortality Rate (2010–2019)

Since 2014, the statistics presented in Table 1 and in Fig. 2 show a slightly increase in the IMR at a national level from 9.85‰ to 11.75‰ in 2019. The neonatal mortality which occurs before 28 days of life is representing the most important part of the IMR (60% in 2019). It is interesting to observe the constantly decreasing trend of birth rate during the same decade, from 21.40‰ to 16.54‰ in 2019.

**Table 1:** National yearly data related to infant mortality

**Year**	**Population**	**Live births**	**Neonatal deaths**	**Post-neonatal deaths**	**Infant deaths**	**Birth rate**	**Neonatal mortality**	**Post-neonatal mortality**	**Infant mortality**
			**0 to 27 days**	**28 days and < 1 year**		**(‰)**	**(‰)**	**(‰)**	**(‰)**
**2010**	15,012,228	321,247	1812	1402	3214	21.40	5.64	4.36	10.00
**2011**	15,266,431	329,510	1842	1218	3060	21.58	5.59	3.70	9.29
**2012**	15,520,973	320,125	1574	1443	3017	20.63	4.92	4.51	9.42
**2013**	15,774,749	296,254	1605	1385	2990	18.78	5.42	4.68	10.09
**2014**	16,027,466	292,395	1566	1313	2879	18.24	5.36	4.49	9.85
**2015**	16,278,844	290,205	1779	1257	3036	17.83	6.13	4.33	10.46
**2016**	16,528,730	281,609	1780	1330	3110	17.04	6.32	4.72	11.04
**2017**	16,776,977	291,582	1907	1405	3312	17.38	6.54	4.82	11.36
**2018**	17,023,408	293,948	2018	1373	3391	17.27	6.87	4.67	11.54
**2019**	17,267,986	285,603	2010	1345	3355	16.54	7.04	4.71	11.75

**Fig. 3:**
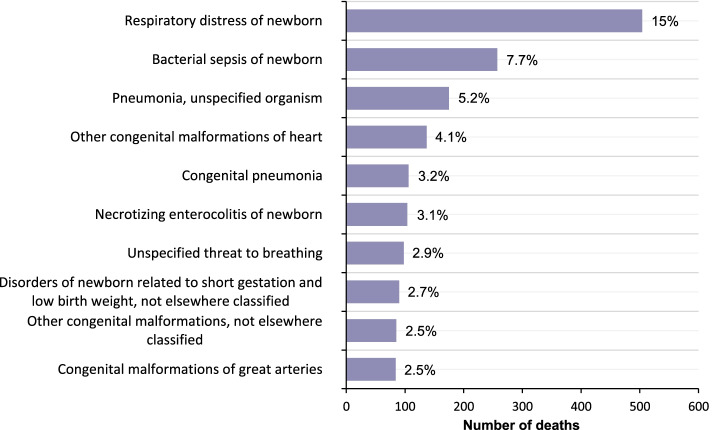
Top ten causes of death in children under one year of age in 2019

Regarding the leading cause of deaths in children under one year old in 2019, of the 3355 children who died 15% (504) died from respiratory distress, 7.7% (257) from bacterial sepsis, 5.2% (175) from pneumonia and 4% (137) from other congenital heart malformations. Figure 3 shows the graph of the top ten causes of mortality in children under one year of age for 2019.

**Fig. 4:**
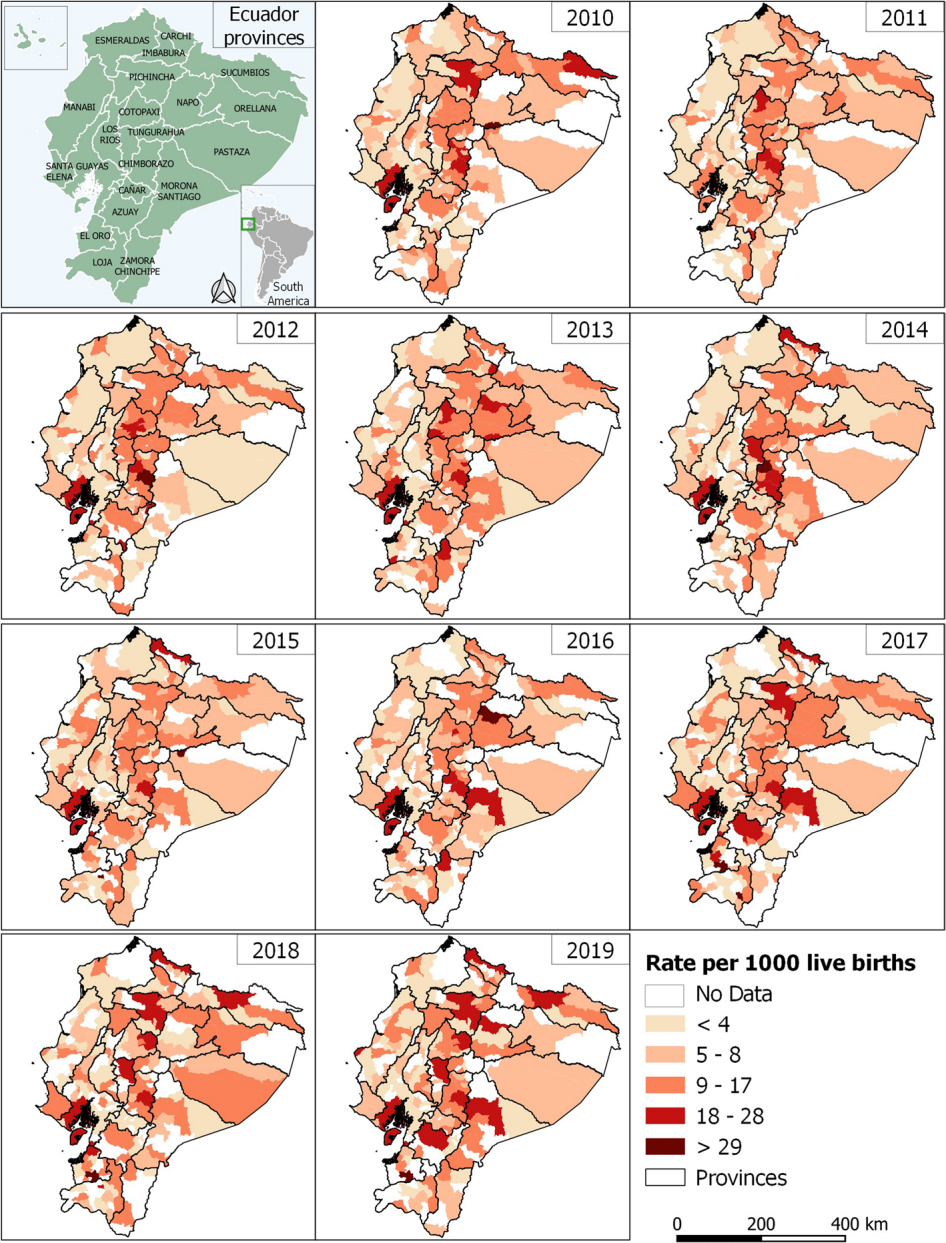
Provinces of Ecuador and Infant Mortality Rate by municipality from 2010 to 2019

**Fig. 5:**
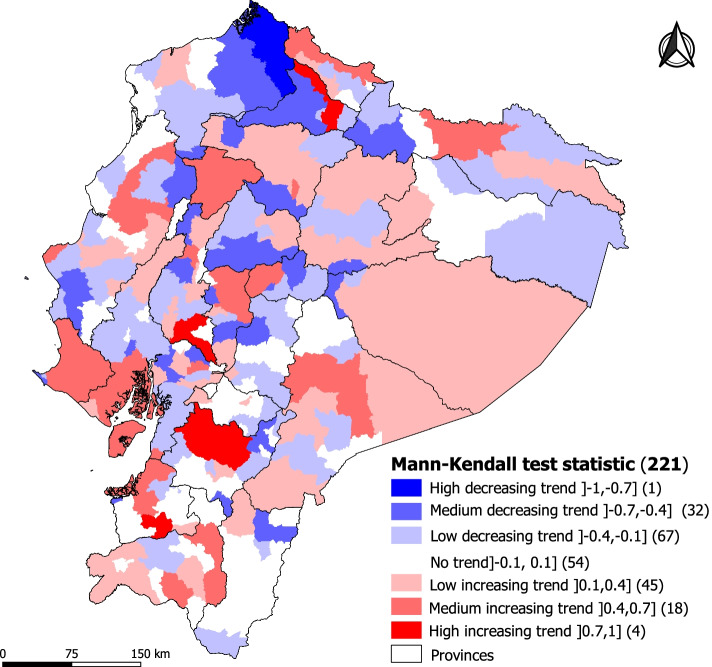
Time trend map of Mann–Kendall (Tau) from 2010 to 2019

Figures 4 and 5 shows the spatial distribution of the incidence rates of mortality in children under one year of age and the temporal trends analyzed by the Mann–Kendall method in the 221 cantons of continental Ecuador.

The trends show that the rates are not spatially constant. At the regional level, there is a slow increase in IMR rates, mainly in the highlands and the Amazon. In the highlands, the cantons with the highest IMR rates are Tulcán (21.67‰), Guaranda (17.86‰) and Cuenca (19.44‰) with medium and high growth trends, respectively, and Latacunga (20.65‰) and Quito (18.77‰) with low growth trends. Similarly, the canton of Guamote (17.34‰) has an IMR above the threshold; however, this trend is steadily decreasing over time.

In the Amazon, of the 41 cantons, 15 maintain an increasing trend between medium and low, however, the cantons of Lago Agrio and Morona are the only ones with a medium increasing trend and with rates above the threshold (20.6‰ and 24.05‰ respectively).

The cantons with the highest growth trends were Piñas, Cuenca, Ibarra and Babahoyo, a particular case on the coast is the Piñas, where the rate increased from 0‰ in 2010 to 157.77 ‰ in 2019 per 1000 live births, making it the canton with the highest increasing trend in the entire country. Another important aspect to highlight within this region is that the cantons Manta and Guayaquil have IMRs of 21.13 ‰ and 21.38 ‰ above the established threshold and with an average upward trend.

The global spatial autocorrelation analysis indicates that in 2010 the value of the Moran index is 0.1485 which is not very high. In 2019 the global Moran index is -0.034 (close to 0) which reflects randomness in the distribution of IMR in the cantons of continental Ecuador.

**Fig. 6:**
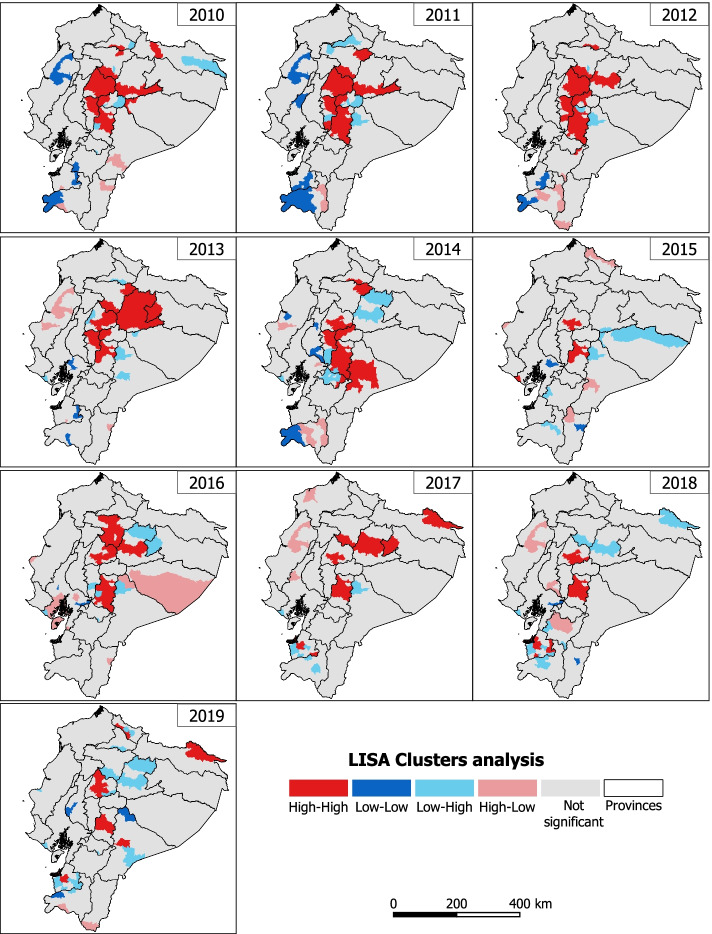
Univariate Local Indicators of Spatial Association by municipality from 2010 to 2019

Through the spatial distribution analysis (Fig. 6), it can be observed that, during the 10 years of the study, most of the high-high geographic clusters (hot spots) are concentrated in the central highlands, which are decreasing over time, until 2019 where they are found in the provinces of Carchi, Chimborazo, Cotopaxi, El Oro, Sucumbíos and Morona Santiago. On the other hand, low-low cold spots appear sporadically in Loja, Los Ríos and Morona Santiago.

Finally, eight priority cantons can be identified, whose IMRs in 2019 are above the selected threshold and whose trends remain increasing over the last 10 years. Table 2 presents the priority cantons in ascending order according to the frequency of high-high clusters. Of these cantons, four belong to the highlands region, two to the coastal region and two to the Amazon region.

**Table 2:** Municipalities with higher risk concerning infant mortality

						**Mann Kendall 2010–2019**	
**Province**	**Region**	**Municipality**	**Infant deaths 2019**	**IMR 2019 (**‰**)**	**Repeated hotspot**	**Tau**	***p*-value**
Bolívar	Highlands	Guaranda	29	17.86	5	0.64	0.0073
Morona Santiago	Amazon	Morona	29	24.05	1	0.69	0.0123
El Oro	Coastal	Piñas	68	157.77	0	0.84	0.0004
Azuay	Highlands	Cuenca	179	19.44	0	0.80	0.0030
Imbabura	Highlands	Ibarra	56	16.74	0	0.73	0.0024
Carchi	Highlands	Tulcán	32	21.67	0	0.60	0.0318
Guayas	Coastal	Guayaquil	994	21.38	0	0.56	0.0200
Sucumbíos	Amazon	Lago Agrio	50	20.60	0	0.56	0.0491

Note: Complete list in supplementary table 1

## Discussion

IM reflects the health status, human development and effectiveness of health systems in a country/region [9, 34]. In this study, we spatially analyzed the evolution of IM as a function of time (annual IMR) and space (trend map and clusters) in Ecuador. We found that the IMR has remained at low levels (see Table 1, Fig. 2), but from 2014 it began to increase until registering 11.75‰ in 2019, neonatal deaths accounted for more than 50% of IM in each year of study and the first cause of death in 2019 was respiratory difficulties (15%). These results are consistent with previous studies, which evidenced that worldwide the highest percentage of deaths in children under one year of age is recorded in the neonatal period (40%), so it is suggested to pay more attention to prematurity and asphyxia of newborns in this period [35, 36], because they are preventable and treatable causes [7].

Our findings identified eight priority municipalities (see Table 2), as they registered high IMR values, showed increasing trends over the years and generated spatial clusters. This is consistent with Gupta et al. [15] who identified priority districts in India as having high IMRs that formed spatial clusters (hot spots). Recent research identified municipalities at high risk of IM as showing increasing trends over time [2, 18]. We found that the highlands region has the majority of municipalities with high IMRs, increasing trends and hot spots. A previous study in Ecuador showed that this region registered the highest rates of the three regions [37], and it was agreed that the IM profile was mainly due to congenital anomalies (Q00-Q99) and diseases of the respiratory system (J00-J99), so it is suggested to redouble efforts to improve the quality of obstetric and neonatal care, essential to prevent and treat these child health problems in a timely manner [38].

Of the eight municipalities, Guaranda, Morona, Piñas and Lago Agrio have the highest percentage of population living in rural areas [39]; these municipalities face unfavorable social and economic conditions, including poverty, literacy, and marginalization of ethnic groups [40], which are related to IM [41]. This result coincides with studies that indicate that IM increases with rurality, and that risk factors associated with infant death such as poverty, ethnic customs (cooking with firewood inside the home), and maternal obesity are more common in these areas [23, 41, 42]. Therefore, specific strategies could be implemented in these areas to improve the socioeconomic conditions of the population, the coverage and accessibility of health services, or even to improve the registration of deaths and births.

Another important point in this study is that the highest IMR are in the most important urban areas of the country (Quito, Guayaquil and Cuenca) and the trend in these areas is increasing, despite the fact that sanitary conditions and medical assistance are much better than in rural areas; however, it should be taken into account that the information considered was analyzed by municipality of death instead of municipality of residence because this detail is not public for confidential reasons. It would be interesting to measure if this inconvenient causes some bias, increasing the risk in big municipalities with hospital facilities where the death of children is better registered and underestimating the problem in rural municipalities where in fact the health deterioration of the child might have occurred.

The spatial analysis applied provides valuable information for the identification of priority municipalities that require immediate attention with respect to IM in Ecuador. In a country with economic limitations, it is important to spatially focus on problematic zones instead of having a national wide politic.

From a health strategy point of view, the focus should be oriented to preventable deaths, it is to say reducible by immunization, appropriate actions for women during pregnancy, fetal growth childbirth, appropriate actions for the newborn, adequate prevention, diagnosis and early treatment, appropriate Health Care and Promotion actions.

Subsequent studies could focus on the components of IM: early neonatal, late neonatal, post-neonatal; preventable or not preventable, spatial and temporal variability of principal cause of death, social determinants that can be spatially and or timely correlated. The epidemiologic week of death could be an additional refinement to this study although the hypothesis a relation between moment of the year and IM is not obvious.

Our study has data limitations. The quality of birth and death statistics is questionable, since in centers that are not connected (e.g., in the Amazon), physical forms are still used. INEC does not provide an accurate assessment of possible underreporting in some regions, but these vital and mortality data, which are necessary for access to public services, can be considered among the most reliable for a relative study.

## Conclusions

This study provides an interesting methodology that combines techniques in spatial and temporal analysis to prioritize critical areas where public allocation of funds should be concentrated and thus contribute to the reduction of IM, especially in a country with economic constraints such as Ecuador.

Places at high risk of IM should benefit from priority interventions. In this sense, the findings of this research are a basis for decision makers in the formulation of policies focused on priority areas of attention, which registered spatial clusters and increasing temporal trends in the IMR. Knowing the exact geographic location of these areas is an additional advantage, because it would help in the efficient management of resources and would have a more effective local impact.

IM is a key indicator that needs to be monitored very closely in order to react in time before a too important increase becomes difficult to control. The identification of higher risk areas may also allow future studies to refine the identification of factors that increase the probability of a rate increase.

## Supplementary Information

**Additional file 1: Supplementary Table 1.** Infant mortalitiy priorization statistics obtained for the 221 municipalities of Ecuador.

## Data Availability

Raw data are freely available at (open access): https://www.ecuadorencifras.gob.ec/nacidos-vivos-y-defunciones-fetales/ [24]; https://www.ecuadorencifras.gob.ec/defunciones-generales/ [25].

## Abbreviations

IM: Infant Mortality
IMR: Infant Mortality Rate
LISA: Local Indicators of Spatial Association
INEC: National Institute of Statistics and Censuses
