# Supplementary material for: The spatio-temporal dynamics of infant mortality in Ecuador from 2010 to 2019
Source: BMC Public Health. 2022 Oct 1;22:1841. doi: 10.1186/s12889-022-14242-1 (PMC9526949; doi:10.1186/s12889-022-14242-1)
Supplement: Supplementary file 1 — Additional file 1: Supplementary Table 1. Infant mortalitiy priorization statistics obtained for the 221 municipalities of Ecuador. [file 12889_2022_14242_MOESM1_ESM.docx]

Supplementary Table 1. Infant mortalitiy priorization statistics obtained for the 221 municipalities of Ecuador

| Province | Municipality | Infant deaths 2019 | IMR 2019 (‰) | LISA cluster 2019 | Mann Kendall (MK) 2010 - 2019 | MK p_value | MK z_value | Number of years pertaining to high high cluster |
| --- | --- | --- | --- | --- | --- | --- | --- | --- |
| Azuay | Cuenca | 179 | 19.44 | Not significant | 0.800 | 0.003 | 2.963 | 0 |
| Azuay | Giron | 0 | 0 | Not significant | 0.111 | 0.855 | 0.183 | 0 |
| Azuay | Gualaceo | 3 | 3.34 | Not significant | -0.556 | 0.049 | -1.968 | 0 |
| Azuay | Nabon | 0 | 0 | Not significant | 0.089 | 0.775 | 0.286 | 0 |
| Azuay | Paute | 0 | 0 | Not significant | -0.578 | 0.037 | -2.082 | 0 |
| Azuay | Pucara | 1 | 8.85 | Not significant | -0.089 | 0.924 | -0.095 | 0 |
| Azuay | San Fernando | 0 | 0 | Not significant | -0.178 | 0.624 | -0.490 | 0 |
| Azuay | Santa Isabel | 0 | 0 | Not significant | -0.067 | 0.685 | -0.406 | 0 |
| Azuay | Sigsig | 1 | 2.14 | Not significant | -0.200 | 0.592 | -0.537 | 0 |
| Azuay | Oña | 0 | 0 | Not significant | -0.444 | 0.096 | -1.667 | 0 |
| Azuay | Chordeleg | 0 | 0 | Not significant | -0.089 | 0.924 | -0.095 | 0 |
| Azuay | El Pan | 0 | 0 | Not significant | 0.000 | 1.000 | 0.000 | 0 |
| Azuay | Sevilla de Oro | 0 | 0 | Not significant | -0.267 | 0.391 | -0.858 | 0 |
| Azuay | Guachapala | 0 | 0 | Not significant | 0.000 | 1.000 | 0.000 | 0 |
| Azuay | Camilo Ponce Enriquez | 0 | 0 | Not significant | -0.311 | 0.141 | -1.471 | 0 |
| Bolivar | Guaranda | 29 | 17.86 | Not significant | 0.644 | 0.007 | 2.683 | 5 |
| Bolivar | Chillanes | 2 | 8 | Not significant | 0.244 | 0.474 | 0.716 | 0 |
| Bolivar | Chimbo | 1 | 5.81 | Not significant | -0.111 | 0.855 | -0.183 | 0 |
| Bolivar | Echeandia | 0 | 0 | Not significant | -0.489 | 0.070 | -1.812 | 0 |
| Bolivar | San Miguel | 0 | 0 | Not significant | -0.400 | 0.174 | -1.358 | 0 |
| Bolivar | Caluma | 0 | 0 | Not significant | 0.244 | 0.359 | 0.917 | 0 |
| Bolivar | Las naves | 0 | 0 | Not significant | -0.067 | 0.832 | -0.212 | 0 |
| Cañar | Azogues | 17 | 13.11 | Not significant | -0.378 | 0.107 | -1.610 | 0 |
| Cañar | Biblian | 3 | 8.24 | Not significant | -0.178 | 0.653 | -0.449 | 0 |
| Cañar | Cañar | 4 | 4.11 | Not significant | 0.022 | 0.858 | 0.179 | 0 |
| Cañar | La Troncal | 4 | 2.4 | Not significant | 0.111 | 0.858 | 0.179 | 0 |
| Cañar | El Tambo | 1 | 5.62 | Not significant | 0.156 | 0.585 | 0.545 | 0 |
| Cañar | Deleg | 0 | 0 | Low-High | 0.000 | 1.000 | 0.000 | 0 |
| Cañar | Suscal | 0 | 0 | Not significant | -0.044 | 1.000 | 0.000 | 0 |
| Carchi | Tulcan | 32 | 21.67 | Not significant | 0.600 | 0.032 | 2.147 | 0 |
| Carchi | Bolivar | 2 | 10.7 | Not significant | -0.044 | 1.000 | 0.000 | 0 |
| Carchi | Espejo | 0 | 0 | Low-High | -0.333 | 0.271 | -1.100 | 0 |
| Carchi | Mira | 1 | 7.63 | High-High | 0.356 | 0.215 | 1.240 | 0 |
| Carchi | Montufar | 2 | 4.84 | Not significant | -0.200 | 0.592 | -0.537 | 0 |
| Carchi | San Pedro de Huaca | 1 | 8 | Not significant | 0.089 | 0.924 | 0.095 | 0 |
| Cotopaxi | Latacunga | 74 | 20.65 | Not significant | 0.244 | 0.474 | 0.716 | 5 |
| Cotopaxi | La Mana | 2 | 2.17 | Not significant | -0.289 | 0.210 | -1.252 | 0 |
| Cotopaxi | Pangua | 2 | 6.56 | Not significant | -0.600 | 0.012 | -2.504 | 0 |
| Cotopaxi | Pujili | 12 | 11.58 | High-High | -0.511 | 0.074 | -1.789 | 0 |
| Cotopaxi | Salcedo | 2 | 2.3 | Low-High | -0.511 | 0.074 | -1.789 | 0 |
| Cotopaxi | Saquisili | 7 | 16.06 | High-High | -0.200 | 0.592 | -0.537 | 0 |
| Cotopaxi | Sigchos | 3 | 11.24 | High-High | -0.378 | 0.107 | -1.610 | 0 |
| Chimborazo | Riobamba | 46 | 10.95 | Not significant | -0.333 | 0.283 | -1.073 | 0 |
| Chimborazo | Alausi | 9 | 13.85 | Not significant | -0.111 | 0.592 | -0.537 | 0 |
| Chimborazo | Colta | 3 | 8.33 | High-High | -0.422 | 0.152 | -1.431 | 0 |
| Chimborazo | Chambo | 1 | 5.46 | Not significant | -0.267 | 0.415 | -0.815 | 0 |
| Chimborazo | Chunchi | 1 | 4.57 | Not significant | 0.067 | 1.000 | 0.000 | 0 |
| Chimborazo | Guamote | 12 | 17.34 | High-High | -0.022 | 1.000 | 0.000 | 8 |
| Chimborazo | Guano | 4 | 6.33 | Not significant | -0.333 | 0.152 | -1.431 | 0 |
| Chimborazo | Pallatanga | 2 | 10.75 | High-High | 0.022 | 1.000 | 0.000 | 0 |
| Chimborazo | Penipe | 0 | 0 | Not significant | -0.022 | 0.848 | -0.192 | 0 |
| Chimborazo | Cumanda | 2 | 8.77 | Not significant | 0.200 | 0.359 | 0.917 | 0 |
| El Oro | Machala | 68 | 13.19 | Not significant | 0.511 | 0.032 | 2.147 | 0 |
| El Oro | Arenillas | 0 | 0 | Low-High | 0.000 | 0.928 | -0.091 | 0 |
| El Oro | Atahualpa | 0 | 0 | Low-High | 0.022 | 1.000 | 0.000 | 0 |
| El Oro | Balsas | 0 | 0 | Low-High | -0.067 | 0.832 | -0.212 | 0 |
| El Oro | Chilla | 0 | 0 | Not significant | 0.000 | 1.000 | 0.000 | 0 |
| El Oro | El Guabo | 9 | 7.98 | Not significant | 0.600 | 0.012 | 2.504 | 0 |
| El Oro | Huaquillas | 0 | 0 | Not significant | -0.422 | 0.074 | -1.789 | 0 |
| El Oro | Marcabeli | 0 | 0 | Low-High | -0.067 | 0.839 | -0.203 | 0 |
| El Oro | Pasaje | 2 | 1.36 | Not significant | -0.111 | 0.592 | -0.537 | 0 |
| El Oro | Piñas | 68 | 157.77 | Not significant | 0.844 | 0.000 | 3.531 | 0 |
| El Oro | Portovelo | 0 | 0 | Low-High | 0.178 | 0.492 | 0.686 | 0 |
| El Oro | Santa Rosa | 20 | 11.72 | High-High | 0.511 | 0.032 | 2.147 | 0 |
| El Oro | Zaruma | 0 | 0 | Low-High | -0.044 | 0.924 | -0.095 | 0 |
| El Oro | Las Lajas | 0 | 0 | Not significant | -0.067 | 0.832 | -0.212 | 0 |
| Esmeraldas | Esmeraldas | 58 | 11.9 | Not significant | 0.200 | 0.371 | 0.894 | 0 |
| Esmeraldas | Eloy Alfaro | 0 | 0 | Not significant | -0.556 | 0.017 | -2.383 | 0 |
| Esmeraldas | Muisne | 0 | 0 | Not significant | 0.000 | 0.924 | 0.095 | 0 |
| Esmeraldas | Quininde | 5 | 2.13 | Not significant | -0.378 | 0.210 | -1.252 | 0 |
| Esmeraldas | San Lorenzo | 0 | 0 | Not significant | -0.756 | 0.002 | -3.143 | 0 |
| Esmeraldas | Atacames | 0 | 0 | Not significant | -0.244 | 0.283 | -1.073 | 0 |
| Esmeraldas | Rioverde | 1 | 1.59 | Not significant | -0.044 | 1.000 | 0.000 | 0 |
| Esmeraldas | La Concordia | 3 | 2.66 | Not significant | -0.556 | 0.049 | -1.968 | 0 |
| Guayas | Guayaquil | 994 | 21.38 | Not significant | 0.556 | 0.020 | 2.326 | 0 |
| Guayas | Alfredo Baquerizo Moreno | 0 | 0 | Not significant | -0.044 | 1.000 | 0.000 | 0 |
| Guayas | Balao | 0 | 0 | Not significant | -0.178 | 0.505 | -0.667 | 0 |
| Guayas | Balzar | 5 | 4.38 | Not significant | 0.111 | 0.592 | 0.537 | 0 |
| Guayas | Colimes | 1 | 2.55 | Not significant | -0.111 | 0.858 | -0.179 | 0 |
| Guayas | Daule | 4 | 1.36 | Not significant | -0.156 | 0.721 | -0.358 | 0 |
| Guayas | Duran | 6 | 1.34 | Not significant | 0.200 | 0.592 | 0.537 | 0 |
| Guayas | Empalme | 2 | 1.24 | Not significant | 0.178 | 0.653 | 0.449 | 0 |
| Guayas | El Triunfo | 4 | 3.91 | Not significant | 0.244 | 0.283 | 1.073 | 0 |
| Guayas | Milagro | 26 | 6.87 | Not significant | 0.378 | 0.210 | 1.252 | 0 |
| Guayas | Naranjal | 3 | 1.9 | Not significant | -0.289 | 0.210 | -1.252 | 0 |
| Guayas | Naranjito | 3 | 3.43 | Not significant | 0.467 | 0.107 | 1.610 | 0 |
| Guayas | Palestina | 0 | 0 | Not significant | 0.089 | 0.922 | 0.098 | 0 |
| Guayas | Pedro Carbo | 3 | 3.41 | Not significant | -0.378 | 0.107 | -1.610 | 0 |
| Guayas | Samborondon | 2 | 1.82 | Not significant | -0.111 | 0.582 | -0.550 | 0 |
| Guayas | Santa Lucia | 1 | 1.61 | Not significant | -0.289 | 0.207 | -1.262 | 0 |
| Guayas | Salitre | 1 | 0.99 | Not significant | -0.200 | 0.371 | -0.894 | 0 |
| Guayas | San Jacinto de Yaguachi | 3 | 2.38 | Not significant | -0.489 | 0.088 | -1.706 | 0 |
| Guayas | Playas | 6 | 4.48 | Low-High | 0.111 | 0.592 | 0.537 | 0 |
| Guayas | Simon Bolivar | 0 | 0 | Not significant | -0.489 | 0.062 | -1.863 | 0 |
| Guayas | Coronel Marcelino Maridueña | 0 | 0 | Not significant | -0.311 | 0.141 | -1.471 | 0 |
| Guayas | Lomas de Aargentillo | 1 | 2.75 | Not significant | 0.089 | 0.653 | 0.449 | 0 |
| Guayas | Nobol | 0 | 0 | Not significant | -0.422 | 0.062 | -1.865 | 0 |
| Guayas | General Antonio Elizalde | 1 | 4.61 | Not significant | 0.089 | 0.769 | 0.294 | 0 |
| Guayas | Isidro Ayora | 0 | 0 | Not significant | -0.244 | 0.359 | -0.917 | 0 |
| Imbabura | Ibarra | 56 | 16.74 | Not significant | 0.733 | 0.002 | 3.041 | 0 |
| Imbabura | Antonio Ante | 3 | 4.26 | Not significant | -0.222 | 0.530 | -0.629 | 0 |
| Imbabura | Cotacachi | 1 | 1.28 | Not significant | -0.467 | 0.107 | -1.610 | 0 |
| Imbabura | Otavalo | 5 | 2.56 | Low-High | -0.467 | 0.049 | -1.968 | 0 |
| Imbabura | Pimampiro | 1 | 4.35 | Not significant | -0.222 | 0.530 | -0.629 | 0 |
| Imbabura | San Miguel de Urcuqui | 0 | 0 | Not significant | -0.400 | 0.085 | -1.720 | 0 |
| Loja | Loja | 69 | 16.37 | Not significant | 0.644 | 0.020 | 2.326 | 0 |
| Loja | Calvas | 2 | 5.38 | Not significant | 0.444 | 0.059 | 1.886 | 0 |
| Loja | Catamayo | 1 | 1.85 | Not significant | -0.044 | 0.786 | -0.272 | 0 |
| Loja | Celica | 0 | 0 | Not significant | 0.378 | 0.093 | 1.679 | 0 |
| Loja | Chaguarpamba | 0 | 0 | Low-High | -0.133 | 0.624 | -0.490 | 0 |
| Loja | Espindola | 0 | 0 | Not significant | 0.044 | 1.000 | 0.000 | 0 |
| Loja | Gonzanama | 1 | 7.35 | Not significant | 0.156 | 0.456 | 0.746 | 0 |
| Loja | Macara | 3 | 10.14 | High-Low | 0.311 | 0.239 | 1.177 | 0 |
| Loja | Paltas | 1 | 3.46 | Not significant | -0.333 | 0.283 | -1.073 | 0 |
| Loja | Puyango | 0 | 0 | Low-Low | 0.133 | 0.634 | 0.477 | 0 |
| Loja | Saraguro | 0 | 0 | Not significant | -0.089 | 0.928 | -0.091 | 0 |
| Loja | Sozoranga | 0 | 0 | Not significant | 0.067 | 0.832 | 0.212 | 0 |
| Loja | Zapotillo | 0 | 0 | Not significant | 0.111 | 0.693 | 0.394 | 0 |
| Loja | Pindal | 0 | 0 | Not significant | 0.000 | 1.000 | 0.000 | 0 |
| Loja | Quilanga | 0 | 0 | Not significant | 0.111 | 0.672 | 0.424 | 0 |
| Loja | Olmedo | 0 | 0 | Not significant | 0.000 | 1.000 | 0.000 | 0 |
| Los Rios | Babahoyo | 30 | 9.43 | Not significant | 0.733 | 0.002 | 3.041 | 0 |
| Los Rios | Baba | 1 | 1.29 | Not significant | 0.244 | 0.463 | 0.733 | 0 |
| Los Rios | Montalvo | 2 | 4.88 | Not significant | -0.022 | 1.000 | 0.000 | 0 |
| Los Rios | Puebloviejo | 1 | 1.22 | Not significant | -0.200 | 0.371 | -0.894 | 0 |
| Los Rios | Quevedo | 33 | 7.82 | Not significant | 0.422 | 0.074 | 1.789 | 0 |
| Los Rios | Urdaneta | 5 | 8.74 | Not significant | -0.289 | 0.371 | -0.894 | 0 |
| Los Rios | Ventanas | 5 | 3.57 | Not significant | 0.244 | 0.474 | 0.716 | 0 |
| Los Rios | Vinces | 6 | 4.07 | Low-Low | -0.333 | 0.283 | -1.073 | 0 |
| Los Rios | Palenque | 0 | 0 | Not significant | -0.200 | 0.359 | -0.917 | 0 |
| Los Rios | Buena Fe | 7 | 4.17 | Not significant | -0.467 | 0.049 | -1.968 | 0 |
| Los Rios | Valencia | 2 | 2.2 | Not significant | 0.178 | 0.415 | 0.815 | 0 |
| Los Rios | Mocache | 0 | 0 | Not significant | -0.556 | 0.040 | -2.052 | 0 |
| Los Rios | Quinsaloma | 0 | 0 | Not significant | 0.000 | 1.000 | 0.000 | 0 |
| Manabi | Portoviejo | 55 | 10.27 | Not significant | 0.244 | 0.283 | 1.073 | 0 |
| Manabi | Bolivar | 3 | 4.05 | Not significant | 0.044 | 1.000 | 0.000 | 0 |
| Manabi | Chone | 20 | 9.05 | Not significant | 0.556 | 0.049 | 1.968 | 0 |
| Manabi | El Carmen | 1 | 0.47 | Not significant | -0.644 | 0.020 | -2.326 | 0 |
| Manabi | Flavio Alfaro | 1 | 1.8 | Not significant | -0.289 | 0.359 | -0.917 | 0 |
| Manabi | Jipijapa | 2 | 1.65 | Not significant | -0.511 | 0.032 | -2.147 | 0 |
| Manabi | Junin | 1 | 2.95 | Not significant | 0.333 | 0.136 | 1.492 | 0 |
| Manabi | Manta | 109 | 21.13 | Not significant | 0.422 | 0.152 | 1.431 | 0 |
| Manabi | Montecristi | 3 | 2.2 | Not significant | -0.222 | 0.530 | -0.629 | 0 |
| Manabi | Pajan | 3 | 4.66 | Not significant | 0.000 | 0.928 | -0.090 | 0 |
| Manabi | Pichincha | 1 | 2.07 | Not significant | 0.244 | 0.283 | 1.073 | 0 |
| Manabi | Rocafuerte | 0 | 0 | Not significant | -0.133 | 0.526 | -0.634 | 0 |
| Manabi | Santa Ana | 0 | 0 | Not significant | -0.111 | 0.709 | -0.373 | 0 |
| Manabi | Sucre | 5 | 4.89 | Not significant | -0.133 | 0.530 | -0.629 | 0 |
| Manabi | Tosagua | 2 | 2.82 | Not significant | -0.089 | 0.928 | -0.090 | 0 |
| Manabi | 24 de mayo | 0 | 0 | Not significant | -0.111 | 0.855 | -0.183 | 0 |
| Manabi | Pedernales | 3 | 2.04 | Not significant | 0.067 | 0.721 | 0.358 | 0 |
| Manabi | Olmedo | 1 | 5.81 | Not significant | 0.244 | 0.263 | 1.119 | 0 |
| Manabi | Puerto Lopez | 0 | 0 | Not significant | -0.378 | 0.199 | -1.283 | 0 |
| Manabi | Jama | 0 | 0 | Not significant | -0.267 | 0.377 | -0.883 | 0 |
| Manabi | Jaramijo | 1 | 2.3 | Low-High | -0.311 | 0.153 | -1.430 | 0 |
| Manabi | San Vicente | 0 | 0 | Not significant | -0.089 | 0.775 | -0.286 | 0 |
| Morona Santiago | Morona | 29 | 24.05 | Not significant | 0.689 | 0.012 | 2.504 | 1 |
| Morona Santiago | Gualaquiza | 5 | 9.47 | Not significant | 0.333 | 0.283 | 1.073 | 0 |
| Morona Santiago | Limon Indanza | 2 | 9.9 | Not significant | -0.267 | 0.239 | -1.177 | 0 |
| Morona Santiago | Palora | 0 | 0 | Low-Low | -0.022 | 0.855 | -0.183 | 0 |
| Morona Santiago | Santiago | 3 | 15.63 | Not significant | 0.311 | 0.323 | 0.988 | 0 |
| Morona Santiago | Sucua | 7 | 11.9 | High-High | 0.200 | 0.371 | 0.894 | 0 |
| Morona Santiago | Huamboya | 0 | 0 | Not significant | -0.133 | 0.769 | -0.294 | 0 |
| Morona Santiago | San Juan Bosco | 0 | 0 | Not significant | 0.156 | 0.542 | 0.609 | 0 |
| Morona Santiago | Taisha | 2 | 2.63 | Not significant | 0.356 | 0.124 | 1.539 | 0 |
| Morona Santiago | Logroño | 0 | 0 | Low-High | -0.289 | 0.192 | -1.306 | 0 |
| Morona Santiago | Pablo Sexto | 0 | 0 | Not significant | 0.000 | 1.000 | 0.000 | 0 |
| Morona Santiago | Tiwintza | 0 | 0 | Low-High | 0.000 | 1.000 | 0.000 | 0 |
| Napo | Tena | 21 | 12.15 | Not significant | 0.289 | 0.371 | 0.894 | 0 |
| Napo | Archidona | 1 | 1.59 | Low-High | -0.111 | 0.858 | -0.179 | 0 |
| Napo | El Chaco | 0 | 0 | Low-High | 0.244 | 0.456 | 0.746 | 0 |
| Napo | Quijos | 2 | 17.7 | Not significant | 0.200 | 0.359 | 0.917 |  |
| Napo | Carlos Julio Arosemena Tola | 0 | 0 | Not significant | -0.400 | 0.062 | -1.863 | 0 |
| Pastaza | Pastaza | 9 | 4.95 | Not significant | 0.200 | 0.592 | 0.537 | 0 |
| Pastaza | Mera | 0 | 0 | Not significant | -0.444 | 0.057 | -1.901 | 0 |
| Pastaza | Santa Clara | 0 | 0 | Not significant | -0.222 | 0.294 | -1.049 | 0 |
| Pastaza | Arajuno | 1 | 4.41 | Not significant | 0.178 | 0.391 | 0.858 | 0 |
| Pichincha | Quito | 747 | 18.77 | Not significant | 0.333 | 0.283 | 1.073 | 1 |
| Pichincha | Cayambe | 15 | 8.54 | Not significant | -0.156 | 0.474 | -0.716 | 0 |
| Pichincha | Mejia | 4 | 2.83 | Low-High | -0.467 | 0.107 | -1.610 | 0 |
| Pichincha | Pedro Moncayo | 4 | 6.09 | Not significant | -0.289 | 0.371 | -0.894 | 0 |
| Pichincha | Rumiñahui | 6 | 3.89 | Not significant | 0.422 | 0.152 | 1.431 | 0 |
| Pichincha | San Miguel de los Bancos | 1 | 4.02 | Not significant | 0.133 | 0.634 | 0.477 | 0 |
| Pichincha | Pedro Vicente Maldonado | 0 | 0 | Not significant | -0.489 | 0.062 | -1.863 | 0 |
| Pichincha | Puerto Quito | 1 | 2.42 | Not significant | 0.156 | 0.714 | 0.367 | 0 |
| Tungurahua | Ambato | 80 | 13.57 | Not significant | 0.644 | 0.007 | 2.683 | 0 |
| Tungurahua | Baños de Agua Santa | 0 | 0 | Not significant | -0.356 | 0.215 | -1.240 | 0 |
| Tungurahua | Cevallos | 0 | 0 | Not significant | -0.289 | 0.223 | -1.218 | 0 |
| Tungurahua | Mocha | 0 | 0 | Not significant | -0.244 | 0.417 | -0.812 | 0 |
| Tungurahua | Patate | 0 | 0 | Not significant | -0.400 | 0.174 | -1.358 | 0 |
| Tungurahua | Quero | 3 | 10.79 | Not significant | -0.222 | 0.526 | -0.634 | 0 |
| Tungurahua | San Pedro de Pelileo | 2 | 2.1 | Not significant | -0.244 | 0.474 | -0.716 | 0 |
| Tungurahua | Santiago de Pillaro | 0 | 0 | Not significant | -0.511 | 0.074 | -1.789 | 0 |
| Tungurahua | Tisaleo | 0 | 0 | Not significant | -0.511 | 0.028 | -2.200 | 0 |
| Zamora Chinchipe | Zamora | 4 | 7.23 | Not significant | 0.067 | 0.721 | 0.358 | 0 |
| Zamora Chinchipe | Chinchipe | 1 | 5.05 | High-Low | -0.156 | 0.709 | -0.373 | 0 |
| Zamora Chinchipe | Nangaritza | 1 | 7.46 | Not significant | -0.044 | 0.775 | -0.286 | 0 |
| Zamora Chinchipe | Yacuambi | 1 | 8.62 | Not significant | 0.267 | 0.415 | 0.815 | 0 |
| Zamora Chinchipe | Yantzaza | 1 | 1.89 | Not significant | -0.489 | 0.085 | -1.720 | 0 |
| Zamora Chinchipe | El Pangui | 1 | 3.89 | Not significant | 0.000 | 0.928 | 0.091 | 0 |
| Zamora Chinchipe | Centinela del Condor | 0 | 0 | Not significant | -0.156 | 0.672 | -0.424 | 0 |
| Zamora Chinchipe | Palanda | 0 | 0 | Not significant | 0.000 | 1.000 | 0.000 | 0 |
| Zamora Chinchipe | Paquisha | 0 | 0 | Not significant | -0.089 | 0.634 | -0.477 | 0 |
| Sucumbios | Lago Agrio | 50 | 20.6 | Not significant | 0.556 | 0.049 | 1.968 | 0 |
| Sucumbios | Gonzalo Pizarro | 0 | 0 | Not significant | -0.533 | 0.059 | -1.886 | 0 |
| Sucumbios | Putumayo | 1 | 5.62 | High-High | -0.200 | 0.371 | -0.894 | 0 |
| Sucumbios | Shushufindi | 2 | 1.91 | Not significant | -0.333 | 0.152 | -1.431 | 0 |
| Sucumbios | Sucumbios | 0 | 0 | Not significant | -0.200 | 0.396 | -0.848 | 0 |
| Sucumbios | Cascales | 2 | 8.16 | Not significant | 0.067 | 0.721 | 0.358 | 0 |
| Sucumbios | Cuyabeno | 2 | 15.75 | Not significant | 0.267 | 0.243 | 1.167 | 0 |
| Orellana | Orellana | 16 | 7.73 | Not significant | -0.067 | 0.721 | -0.358 | 0 |
| Orellana | Aguarico | 0 | 0 | Not significant | -0.111 | 0.542 | -0.609 | 0 |
| Orellana | La Joya de los Sachas | 0 | 0 | Not significant | -0.244 | 0.283 | -1.073 | 0 |
| Orellana | Loreto | 3 | 4.99 | Not significant | 0.156 | 0.721 | 0.358 | 0 |
| Santo Domingo de los Tsachilas | Santo Domingo | 90 | 10.19 | Not significant | 0.511 | 0.074 | 1.789 | 0 |
| Santa Elena | Santa Elena | 17 | 4.9 | Not significant | 0.422 | 0.074 | 1.789 | 0 |
| Santa Elena | La Libertad | 5 | 2.44 | Not significant | -0.289 | 0.371 | -0.894 | 0 |
| Santa Elena | Salinas | 1 | 0.7 | Not significant | -0.467 | 0.049 | -1.968 | 0 |
| Undelimited area | Las Golondrinas | 0 | 0 | Not significant | -0.111 | 0.525 | -0.636 | 0 |
| Undelimited area | Manga del Cura | 0 | 0 | Not significant | 0.000 | 1.000 | 0.000 | 0 |
| Undelimited area | El Piedrero | 0 | 0 | Not significant | 0.000 | 1.000 | 0.000 | 0 |
